# Characterization and epidemiology of antimicrobial resistance patterns of *Salmonella* spp. and *Staphylococcus* spp. in free-ranging rhesus macaque (*Macaca mulatta*) at high-risk interfaces with people and livestock in Bangladesh

**DOI:** 10.3389/fvets.2023.1103922

**Published:** 2023-01-30

**Authors:** Md. Kaisar Rahman, Mohammad Mahmudul Hassan, Shariful Islam, Melinda K. Rostal, Md. Helal Uddin, Emily Hagan, Mohammed Abdus Samad, Meerjady Sabrina Flora, Jonathan H. Epstein, Ariful Islam

**Affiliations:** ^1^Faculty of Veterinary Medicine, Chattogram Veterinary and Animal Sciences University, Chattogram, Bangladesh; ^2^Institute of Epidemiology, Disease Control and Research (IEDCR), Dhaka, Bangladesh; ^3^EcoHealth Alliance, New York, NY, United States; ^4^School of Veterinary Medicine, Texas Tech University, Amarillo, TX, United States; ^5^Queensland Alliance for One Health Sciences, School of Veterinary Science, The University of Queensland, Brisbane, QLD, Australia; ^6^Antimicrobial Resistance Action Center (ARAC), Bangladesh Livestock Research Institute, Savar, Bangladesh; ^7^Directorate General of Health Services, Dhaka, Bangladesh

**Keywords:** rhesus macaque, antimicrobial resistance, *Salmonella*, *Staphylococcus*, Bangladesh

## Abstract

**Introduction:**

Antimicrobial resistance (AMR) is a growing global health threat for humans and animals. Environmental contamination of antimicrobials from human and domestic animal feces has been linked to AMR in wildlife populations, including rhesus macaques. This study aimed to describe the eco-epidemiology of AMR within *Salmonella* and *Staphylococcus* species isolated from rhesus macaques.

**Methods:**

We followed macaque groups for 4 h per day (2 days) to observe the direct and indirect contact rate and type between macaques and people and livestock. We collected 399 freshly defecated, non-invasive fecal samples from macaques at seven sites in Bangladesh in January–June 2017. Bacterial isolation and identification were conducted using culture, biochemical characteristics, and polymerase chain reaction (PCR). An antimicrobial susceptibility test (AST) for 12 antimicrobials for each organism was conducted using the Kirby–Bauer disc diffusion method.

**Results:**

The overall prevalence of *Salmonella* spp. and *Staphylococcus* spp. in rhesus macaques was 5% (*n* = 18; 95% CI: 3–7%) and 16% (*n* = 64; 95% CI: 13–20%), respectively. All the isolated *Salmonella* spp. and most of the *Staphylococcus* spp. (95%; 61/64; 95% CI: 86.9–99%) were resistant to at least one antimicrobial. The odds of a fecal sample having antimicrobial-resistant *Salmonella* spp (OR = 6.6; CI: 0.9–45.8, *P* = 0.05) and *Staphylococcus* spp. (OR = 5.6; CI: 1.2–26, *P* = 0.02) were significantly higher in samples collected at peri-urban sites than those collected at rural and urban sites. *Salmonella* spp. were most frequently resistant to tetracycline (89%), azithromycin (83%), sulfamethoxazole-trimethoprim (50%), and nalidixic acid (44%). *Staphylococcus* spp. were found to be highly resistant to ampicillin (93%), methicillin (31%), clindamycin (26%), and rifampicin (18%). Both bacterial species produced colonies with multidrug resistance to up to seven antimicrobials. Direct and indirect contact rates (within 20 m for at least 15 min) and resource sharing between macaques and people were higher in urban sites, while macaque-livestock contact rates were higher in rural sites.

**Discussion:**

The study shows that resistant microorganisms are circulating in rhesus macaque, and direct and indirect contact with humans and livestock might expand the resistant organisms.

## 1. Introduction

Antimicrobial-resistant bacteria represent a critical health threat to people, domestic animals, and wildlife. Antimicrobial resistance (AMR) is recognized as a significant emerging public health concern by the World Health Organization (WHO) and represents a significant challenge to human and veterinary medicine due to the therapeutic failure of life-saving treatments (1). Natural ecosystems are believed to be contaminated with antimicrobial-resistant bacteria due to excessive antimicrobial use in clinical and agriculture settings (2). Antibiotics are used for therapeutic, prophylactic, and growth promotion purposes in livestock and poultry (3–5). Further, the application of manure as fertilizer for crop growth has been associated with AMR, regardless of whether the animals that produced the manure were treated with antibiotics, because the manure enriches the environment, allowing the growth of resident soil bacteria that harbor AMR elements (6). In Bangladesh, it is common for people to use antimicrobials without consulting with a registered doctor or veterinarian to treat themselves or other people and animals. Indiscriminate use of antimicrobials in multiple health sectors accelerates the development of antimicrobial resistance in bacteria. A One Health approach is important to identify the human, animal, and environmental factors associated with increasing levels of AMR (7).

Globalization has driven AMR to become a global problem. It is known that the movement of people and animals can permit the rapid spread of resistant pathogens (8). Asia is considered an epicenter of AMR, especially in densely populated South Asian countries such as Bangladesh and India (9). Multidrug-resistant genes are prevalent in many commensal and environmental bacteria, including *Escherichia coli, Klebsiella pneumonia, Salmonella, Enterococcus*, and *Staphylococcus aureus* (10). Multiple studies have revealed that bacterial resistance genes, which are frequently detected in human or animal microbiota, are spreading to environments and wild animals where antimicrobials have not previously been used (11).

Antimicrobial-resistant microorganisms in wildlife are frequently detected (12). Bacteria resistant to antimicrobials have been detected in various wild animal species (13) and wild birds (14). Wildlife has been implicated as a potential reservoir of resistant bacteria and resistance genes (15). Wild animals are rarely treated with antimicrobials. Thus, the source of antimicrobial-resistant bacteria in wildlife is not clear. Some proposed transmission routes are the consumption of human feces, household and kitchen waste, and sewage water (16). Several studies in Bangladesh have already proved the presence of resistant microorganisms in household water supply (17) and environmental effluents (7, 18). Potential exposure to resistant bacteria likely depends on host species, as some species have more frequent contact with humans, human landscapes, or domestic animals than others (19). Antimicrobial-resistant bacteria have been found in 54% of wild rodents living in proximity to livestock harbored bacteria resistant to at least one antimicrobial agent (20).

Rhesus macaques (*Macaca mulatta*) are synanthropic, thriving in human-altered environments, allowing them to become one of the most widely distributed and successful primates (21). Macaques are distributed throughout Bangladesh, living in urban, rural, and forested/protected areas. Feeroz et al. (22) described non-human primate habitats in the northeastern and southeastern parts of Bangladesh. One study estimated that there were 5,313 macaques in Bangladesh, with group sizes varying from 10 to 78 animals (23).

It has been estimated that up to 61% of monkeys and great apes in sub-Saharan Africa (Gabon and Ivory Coast) are infected with *Staphylococcus aureus* (24). Among captive rhesus macaques, infections with *Staphylococcus* spp. in the wild non-human primate are also common, with a prevalence of 39% rhesus macaques in the Netherlands (25) and 23.6% monkeys in Wisconsin, USA (26). Other potential human bacterial pathogens have been identified in multiple primate species. This includes human-habituated mountain gorillas (*Gorilla gorilla beringei*) in Uganda, with prevalences of 19, 13, and 6% for *Campylobacter* spp., *Salmonella* spp., and *Shigella* spp., respectively (27). Evidence of *Escherichia coli* sharing between people, domestic animals, and great apes has been reported in densely human-populated areas of western Uganda. Within Uganda, habituated groups of wild apes are visited daily by researchers and tourists [e.g., chimpanzees (28) and mountain gorillas at Bwindi Impenetrable National Park (29)].

Several studies have identified antimicrobial-resistant bacteria in non-human primates. A study indicated that 75% of *Staphylococcus aureus* isolates from captive rhesus macaques in the United States were resistant to methicillin/oxacillin (30). *Campylobacter* spp. were isolated from 15, 36, and 67% of cynomolgus macaques (*Macaca fascicularis*) in China, Cambodia, and Indonesia, respectively, and resistance to tetracycline, ciprofloxacin, and erythromycin was prevalent in China (80%) and Cambodia (75%), as well as in Indonesia (100%) (31). Rolland et al. (16) found that non-human primates (baboons) feeding on human garbage and in contact with human feces had significantly greater levels of antibiotic-resistant gut bacteria than their counterparts.

In wildlife, direct and indirect interspecies contacts are likely critical routes of transmission of antimicrobial-resistant bacteria (32). These routes include consuming food and water contaminated by antimicrobial residues or resistant bacteria within or through indirect contact with resistant bacteria in a contaminated environment (15). Wildlife at zoos and safari parks may potentially be administered antimicrobials directly. Areas with high rates of interactions between people or livestock and non-human primates are recognized as potential drivers of inter-species pathogens transmission (33).

AMR dynamics has a potential role in human, animal, and environmental health by increasing zoonotic diseases and microorganisms, but knowledge of AMR research in wildlife is limited. It has been hypothesized that the occurrence of antibiotic-resistant strains in untreated, free-ranging wild animals is a consequence of bacterial transmission with people or domestic animals which have undergone antibiotic treatment. Here we evaluated the prevalence of two bacteria (*Salmonella* spp. and *Staphylococcus* spp.) and their antimicrobial resistance levels in rhesus macaques from different districts and habitats.

## 2. Materials and methods

### 2.1. Ethical approval

Ethical approval was approved by Chattogram Veterinary and Animal Sciences University-Animal Experimentation Ethics Committee (AEEC), Chattogram, Bangladesh [permit number: CVASU/Dir (R&E) AEEC/2015/751] to conduct the study. With the help of a defined protocol, we assured the animal ethics and animal safety as well as the safety of working personnel in both field and laboratory throughout the whole study period.

### 2.2. Study areas, study design, and period

**Figure 1:**
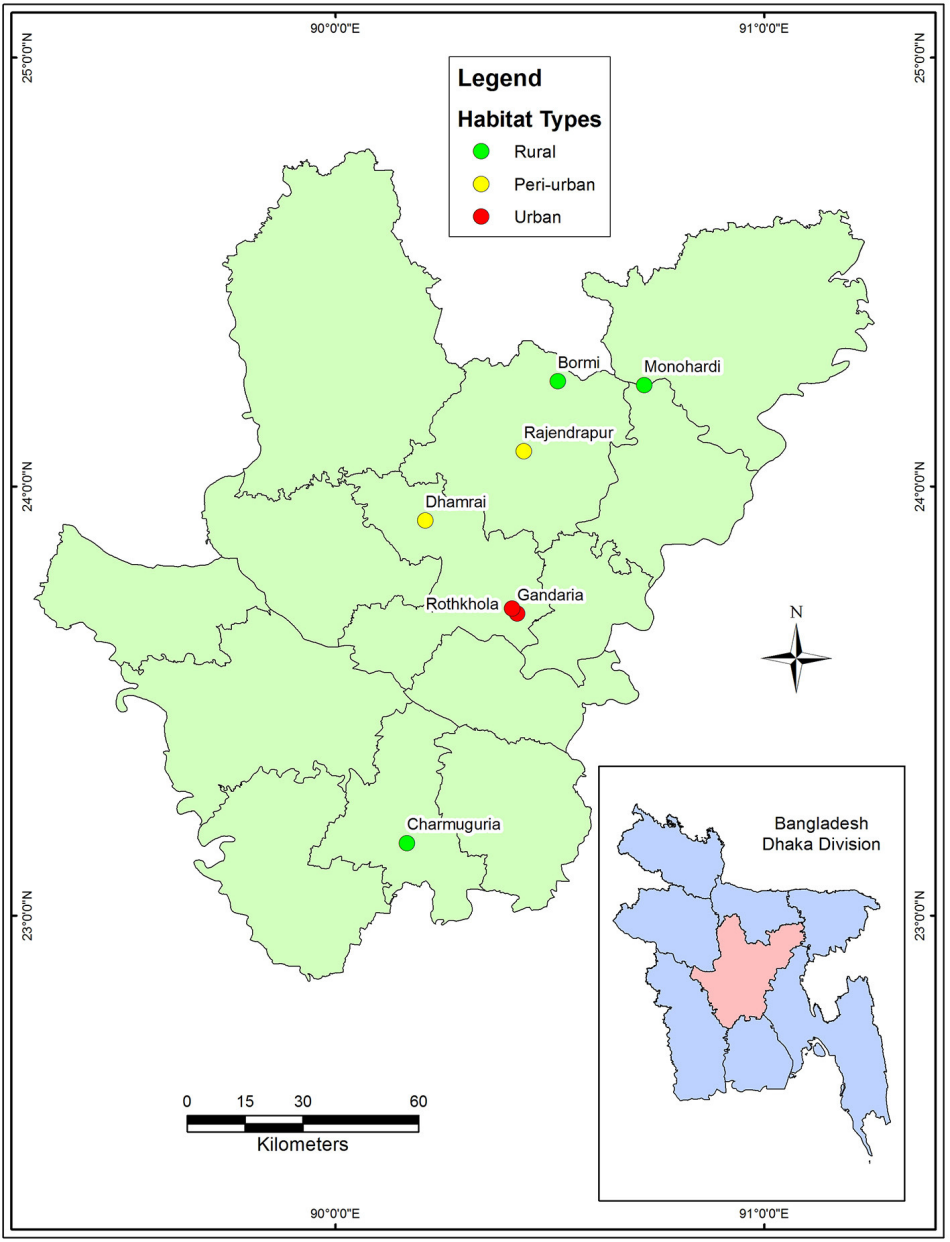
Map showing the sampling locations.

A cross-sectional study was conducted from January to June 2017. The macaque population in Bangladesh is distributed among urban, peri-urban, and rural habitats where they live close to human settlements. We selected seven sites from four districts: Dhaka (Gendaria, Rothkhola, and Dhamrai), Madaripur (Charmuguria), Gazipur (Rajendrapur and Bormi), and Narshingdi (Monohardi) (Figure 1). The distribution of rhesus macaque populations in Dhaka, Madaripur, Narshingdi, and Gazipur districts, where temples and shrines had been built from the earliest stages of civilization (34). Nevertheless, the macaque population is prevalent in these regions, thus we chose these sampling locations for our study. The sampling map was plotted using the spatial analyst tool of ArcGIS (ArcMap, version 10.2, Environmental Systems Research Institute, Redlands, California, CA, USA).

### 2.3. Sample collection

The required sample size was calculated to be 385 using the formula given by Daniel and Cross (35). We collected a total of 399 samples. Samples were collected proportionally (>60%) from each site's macaque population (Table 1). Macaques were baited by provisioning bread and bananas before sample collection. We observed defecation and immediately collected fecal samples (~1 gm) using convenience sampling. All samples were collected within 30 min to avoid re-sampling the same individual. Four trained personnel collected the samples and identified the individual animal's sex and age. Each monkey was seen defecating during the sampling period by two trained persons. To prevent duplication sampled monkey was continuously observed until the sampling was completed (21). Confirmation of the age was also done by the fecal lobe diameter (27). Fecal samples were collected by sterile swab and placed in a falcon tube (15 ml) containing buffered peptone water (Oxoid, Basingstoke, Hampshire, UK) for *Salmonella* spp. (36) and Mueller-Hinton broth for *Staphylococcus* spp. (37). Falcon tubes were placed in the ice box, and within 24 hours, they were sent to Bangladesh Livestock Research Institute (BLRI), Savar, for laboratory analysis.

**Table 1:** Frequency distribution of samples from different location.

**District**	**Site**	**Group**	**Population size**	**Sample number**	**Proportion**
Dhaka	Gandaria	i	57	35	61.40
		ii	55	35	63.64
	Rothkhola	i	56	35	62.50
		ii	54	35	64.81
	Dhamrai	i	53	33	62.26
		ii	51	33	64.71
Madaripur	Charmuguria	i	41	25	60.98
		ii	32	20	62.50
Gazipur	Rajendrapur	i	36	22	61.11
		ii	34	22	64.71
	Bormi	i	40	26	65.00
		ii	38	25	65.79
Narshingdi	Monohardi	i	53	33	62.16
		ii	32	20	62.50

### 2.4. Data collection

We made field observations to collect data on population structure age (adult, sub-adult, and infant), and sex and ecological data: types of habitats (rural, urban and peri-urban), season [winter (January to March), and summer (April to June)] and GPS coordinates. We also observed the interactions rhesus macaques had with people, domestic animals, and sampling sites for 2 h in the morning and 2 h in the evening for 2 days (a total of 8 h). We identified the frequency and type of both direct contact and indirect contact. Direct contact was defined as any type of inter-species touching/contact, biting, and scratching. Whereas, indirect contact was recorded if the macaques remained within 20 meters of another species for at least 15 min.

### 2.5. Laboratory testing

#### 2.5.1. Isolation and identification of bacteria

*Salmonella* spp. was cultured in xylose lysine deoxycholate (XLD) agar (producing a black centered colony) and on brilliant green agar (BGA) media (producing red-colored colony) (38), *Staphylococcus* spp was cultured on mannitol salt agar producing yellow color and cocci-shaped gram-positive clusters (37). All the positive samples were confirmed using biochemical assays: triple sugar iron (TSI) agar slant (39), and carbohydrate fermentation test (40) were used for *Salmonella* spp., and the coagulase (41) and catalase test (42) were used for *Staphylococcus* spp. Finally, PCR was conducted for final confirmation of a bacterial genus *Salmonella* with Salmonella genus-specific primers ST-11 (5′ -AGCCAACCATTGCTAAATTGGCGCA-3′) and ST-15(5′-TGGTAGAAATTCCCAGCGGGTACTG-3′ described by Gouws et al. (43) and *Staphylococcus* with Staphylococcus genus-specific primers TStaG422 (5′-GGC CGT GTT GAA CGT GGT CAA ATC A-3′) and TStag765 (5′-TIA CCA TTT CAG TAC CTT CTG GTA A-3′) described by Martineau et al. (44).

#### 2.5.2. Antimicrobial susceptibility test

A panel of 12 antibiotics was used for each organism: chloramphenicol (CPL) (30 μg), gentamicin (GEN) (10 μg), sulfamethoxazole-trimethoprim (SXT) (25 μg), and tetracycline (TET) (30 μg) were used for both genera. In addition, amoxicillin-clavulanic acid (AMC) (30 μg), azithromycin (AZM) (15 μg), cefixime (CFM) (5 μg), cefotaxime (CTX) (30 μg), ceftriaxone (CTR) (30 μg), ciprofloxacin (CIP) (5 μg), imipinem (IPM) (10 μg), nalidixic acid (NA) (30 μg) were used for *Salmonella* spp. For *Staphylococcus*, we used: ampicillin (AMP) (10 μg), clindamycin (CDA) (2 μg), linezolid (LZD) (30 μg), methicillin (MET) (5 μg), oxacillin (OXA) (1 μg), rifampicin (RMP) (5 μg), streptomycin (STP) (10 μg), tigecyclin (TGC) (15 μg). We used the Kirby-Bauer disc diffusion method for antimicrobial susceptibility testing (45). Zone diameters were calculated and interpreted as resistant, intermediate, and sensitive according to the size of inhibition following the Clinical and Laboratory Standards Institute (CLSI) guideline, 25th edition (46).

### 2.6. Statistical evaluation

Field and laboratory data were entered into MS Excel (Excel 2013, Microsoft Corporation, USA). After checking data integrity, it was exported to STATA/IC13 (StataCorp 4905, Lakeway Drive, College Station, Texas 77845, USA) for epidemiological analysis. The prevalence of the bacteria and antimicrobial-resistant bacteria were expressed as a percentage with a 95% confidence interval (CI). If any microorganism was found to be resistant to a single antimicrobial, it was considered resistant. Antimicrobial susceptibility was expressed as a percentage according to resistance, intermediate, and sensitivity. Variables were assessed for collinearity. A univariate Chi-square test was done to identify potential risk factors for the presence of AMR. The significant risk factors (*P* < 0.2) were included in multiple logistic regression models, but the district was omitted for *Salmonella* spp. due to collinearity. Model adequacy was verified by the lowest AIC after dropping variables from the model. Confounders were confirmed by observing the variation in the coefficient (β > 10%). The final model was assessed using a receiver operating characteristic curve (ROC) and goodness of fit tests (47). The results were expressed as odds ratios (ORs) and 95% CI.

## 3. Results

We sampled 399 macaques. The overall prevalence of *Salmonella* spp. and *Staphylococcus* spp. in macaques were 5% (*n* = 18; 95%CI: 2.7–7.1%) and 16% (*n* = 64; 95%CI: 12.6–20%), respectively. All of the *Salmonella* spp. and 95% of the *Staphylococcus* spp. (*n* = 61; 95%CI: 86.9–99%) isolated in the study were resistant to at least one antimicrobial.

### 3.1. Descriptive statistics for macaques carrying antimicrobial-resistant *Salmonella* spp.

Among all macaques, the prevalence of resistant *Salmonella* spp. was highest in macaques in the Dhaka District (*n* = 15; 7.3%; 95%CI: 4.1–11.7), whereas the lowest prevalence (*n* = 1; 1.1%; 95%CI: 0.03–5.7) was found in Gazipur (see Table 2 for all descriptive statistics). Habitat, season, and age were included in the multivariable regression, and none of the variables were significant in the multiple logistic regression (Table 2).

**Table 2:** Frequency distribution of resistant *Salmonella* spp. in rhesus macaque of Bangladesh.

**Variables**	**Categories**	**Resistant** ***Salmonella*** **spp**.			**Multiple logistic regression**		
		***n*** **(%)**	**95% CI**	***P*** **(**χ^2^**-test)**	**OR**	**95% CI**	***P***
District^†^	Dhaka (206)	15 (7.3)	4.1–11.7	0.05			
	Gazipur (95)	1 (1.1)	0.03–5.7				
	Madaripur (45)	1 (2.2)	0.06–11.8				
	Narshingdi (53)	1 (1.9)	0.05–10.1				
Habitat	Rural (149)	2 (1.3)	0.16–4.7	0.03	Ref		
	Peri-urban (110)	5 (4.6)	1.5–10.3		6.6	0.9–45.8	0.05
	Urban (140)	11 (7.9)	3.9–13.6		4.2	0.9–20.8	0.07
Seasons	Winter (242)	7 (2.9)	1.2–5.9	0.05	Ref		
	Summer (157)	11 (7.1)	3.6–12.2		3.1	0.6–15.2	0.16
Age	Juvenile (105)	2 (1.9)	0.2–6.7	0.06	Ref		
	Sub-adult (113)	3 (2.7)	0.6–7.6		1.3	0.2–7.7	0.80
	Adult (181)	13 (7.2)	3.9–11.9		3.8	0.8–17.4	0.08
Sex	Male (174)	7 (4.0)	1.6–8.1	0.68			
	Female (225)	11 (4.9)	2.5–8.6				

^†^Excluded from analysis due to collinearity with habitat.

### 3.2. Descriptive statistics and risk factors for macaques carrying antimicrobial-resistant *Staphylococcus* spp.

In univariate analysis, only sex did not meet the selection criteria to be included in the multivariable analysis. In the multivariable analysis, we found that macaques sampled in Dhaka (OR = 11.1, CI: 1.2–106.9, *P* = 0.03), Gazipur (OR = 9.3, CI: 1.1–73.1, *P* = 0.03) and Madaripur (OR = 8.5, CI: 1.1–69.2, *P* = 0.04) had significantly higher odds of carrying antimicrobial resistant *Staphylococcus* spp, compared to animals living in Narshingdi (Table 3). Additionally, macaques sampled in a peri-urban habitat (OR = 5.6, CI: 1.2–26, *P* = 0.02) and those that were sub-adults (OR = 2.1, CI: 1.1–4.0, *P* = 0.03) had higher odds of infection (Table 3).

**Table 3:** Frequency distribution of resistant *Staphylococcus* spp. in rhesus macaque of Bangladesh.

**Variables**	**Categories**	**Resistant** ***Staphylococcus*** **spp**.			**Multiple logistic regression**		
		***n*** **(%)**	**95% CI**	***P*** **(**χ^2^**-test)**	**OR**	**95% CI**	***P***
District	Dhaka (206)	31 (15.1%)	10.5–20.7	0.13	11.1	1.2–106.9	0.03
	Gazipur (95)	19 (20%)	12.5–29.4		9.3	1.1–73.1	0.03
	Madaripur (45)	8 (17.8%)	08–32.1		8.5	1.1–69.2	0.04
	Narshingdi (53)	3 (5.7%)	01.2–15.7		Ref		
Habitat	Rural (149)	21 (14.1%)	08.9–20.7	0.01	5.0	0.8–31.6	0.08
	Peri-urban (110)	26 (23.6%)	16.1–32.7		5.6	1.2–26	0.02
	Urban (140)	14 (10%)	05.6–16.2		Ref		
Seasons	Winter (242)	46 (19.1%)	14.3–24.5	0.01	0.5	0.09–2.2	0.33
	Summer (157)	15 (9.6%)	05.4–15.3		Ref		
Age	Juvenile (105)	16 (15.2%)	08.9–23.6	0.08	1.2	0.6–2.5	0.57
	Sub-adult (113)	24 (21.2%)	14.1–29.9		2.1	1.1–4.0	0.03
	Adult (181)	21 (11.6%)	07.3–17.2		Ref		
Sex	Male (174)	28 (16.1%)	10.9–22.4	0.69			
	Female (225)	33 (14.7%)	10.3–19.9				

### 3.3. AMR patterns and multidrug resistance of *Salmonella* spp. in rhesus macaque

**Figure 2:**
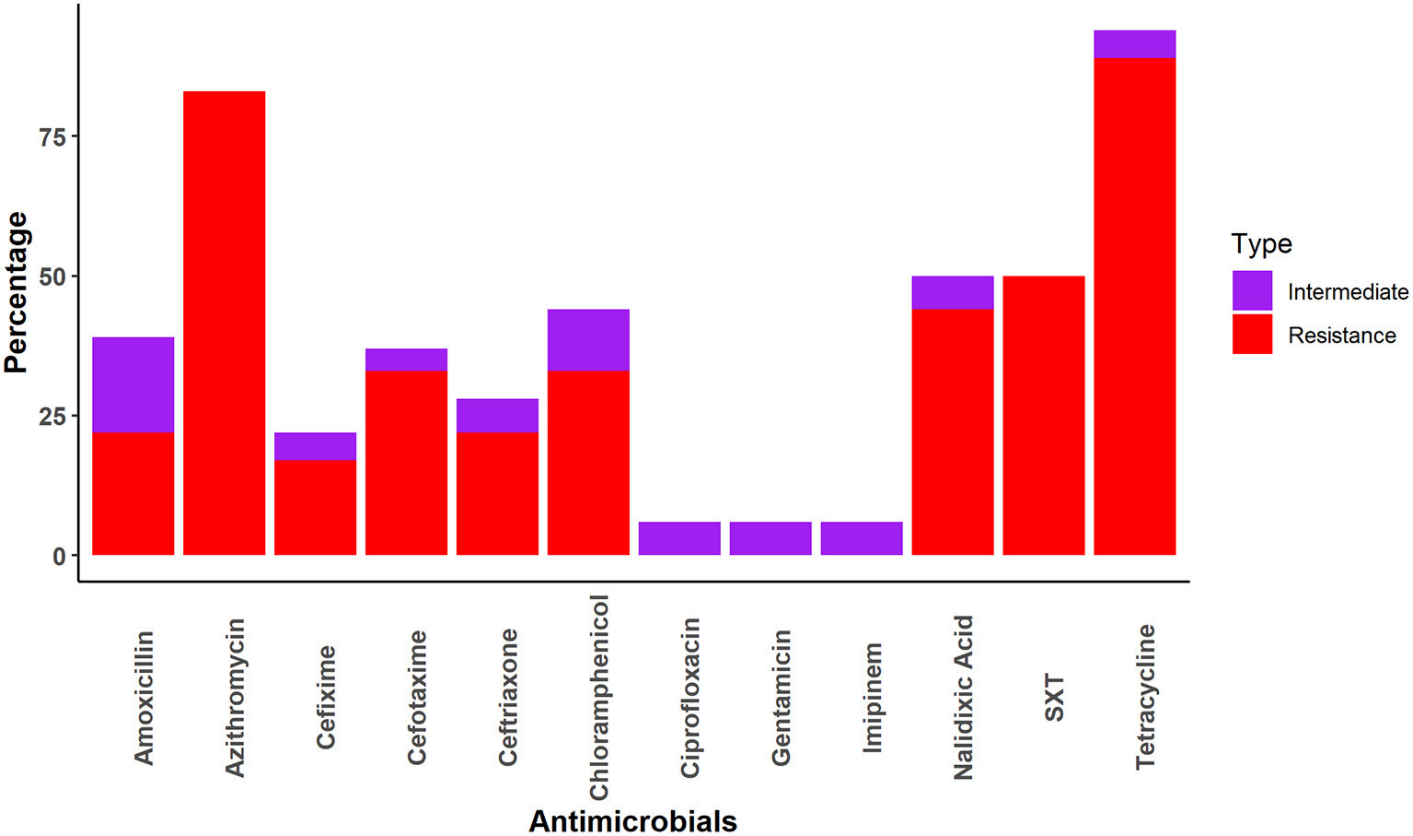
AMR pattern of *Salmonella* spp.

*Salmonella* spp. was most frequently resistant to tetracycline (TET) (89%), followed by azithromycin (AZM) (83%) and cefixime (17%). However, *Salmonella spp*. demonstrated no resistance to ciprofloxacin, gentamicin, or imipenem (Figure 2).

A total of seven combinations of drugs were found to be resistant to *Salmonella* spp. Two combinations of multiple drugs were found most frequently for *Salmonella* spp.; AZM-TET (27.8%; 95%CI: 9.7–53.5) and AZM-CPL-NA-SXT-TET (22.2%; 95%CI: 6.4–47.6). All other combinations of multidrug resistance were detected in a single sample (Table 4).

**Table 4:** Multidrug resistance patterns of *Salmonella* spp.

**Number of drugs**	**Resistance patterns**	**Frequency**	**Percentage (%)**	**95% CI**	**Multidrug resistance**
One drug	TET	1	5.5	0.1–27.3	
Two drugs	AZM-TET	5	27.8	9.7–53.5	
Three drugs	AZM-CTX-TET	1	5.5	0.1–27.3	Yes
	CFM-CTX-CTR	1	5.5	0.1–27.3	
Four drugs	CFM-CTX-CTR-SXT	1	5.5	0.1–27.3	
Five drugs	AMC-AZM-CTX-CTR-TET	1	5.5	0.1–27.3	Yes
	AMC-AZM-NA-SXT-TET	1	5.5	0.1–27.3	Yes
	AZM-CPL-NA-SXT-TET	4	22.2	6.4–47.6	Yes
Six drugs	AZM-CTR-CPL-NA-SXT-TET	1	5.5	0.1–27.3	Yes
Seven drugs	AMC-AZM-CFM-CTX-NA-SXT-TET	1	5.5	0.1–27.3	Yes
	AMC-AZM-CTX-CPL-NA-SXT-TET	1	5.5	0.1–27.3	Yes

AMC, amoxicillin-clavulanic acid; AZM, azithromycin; CFM, cefixime; CTX, cefotaxime; CTR, ceftriaxone; CPL, chloramphenicol; NA, nalidixic acid; SXT, sulfamethoxazole-trimethoprim; TET, tetracycline.

### 3.4. AMR patterns and multidrug resistance of *Staphylococcus* spp. in rhesus macaque

**Figure 3:**
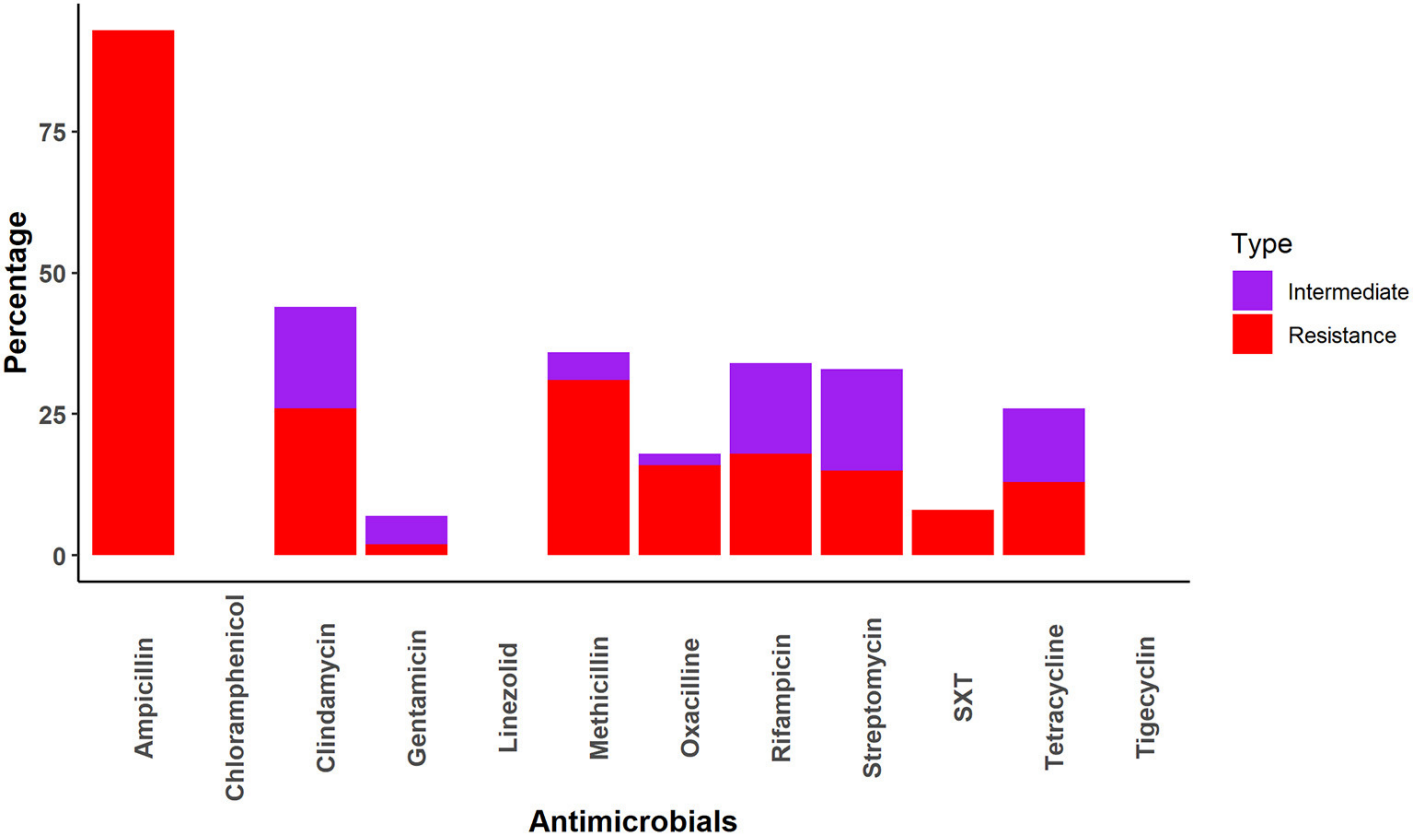
AMR patterns of *Staphylococcus* spp.

All *Staphylococcus* samples were resistant to at least one drug. The *Staphylococcus* samples were most frequently resistant to ampicillin (93%), followed by methicillin (31%), clindamycin (26%), rifampicin (18%), oxacillin (16%), streptomycin (15%), tetracycline (13%) and sulfamethoxazole-trimethoprim (8%). No resistance was identified against chloramphenicol, linezolid, or tetracycline (Figure 3).

Nearly half of the *Staphylococcus* cultures were resistant to only one drug (AMP [42.6%] and MET [3.3%]; Table 5). Four samples had resistance patterns of each AMP-CDA (6.5%) and AMP-CDA-MET (6.5%) combination (Table 5). Multidrug combinations of one, two, three, four, five, six, and seven drugs were identified (Table 5); however, most combinations were only found in a single sample.

**Table 5:** Multidrug resistance pattern of *Staphylococcus* spp.

**Number of drugs**	**Resistance patterns**	**Frequency**	**Percentage**	**95% CI**	**Multidrug resistance**
One drug	AMP	26	42.6	30.0–55.9	
	MET	2	3.3	0.4–11.3	
Two drugs	AMP-CDA	4	6.5	1.8–15.9	
	AMP-RMP	2	3.3	0.4–11.3	
	AMP-STR	1	1.6	0.04–8.8	
	AMP-TET	1	1.6	0.04–8.8	
	AMP-SXT	2	3.3	0.4–11.3	
	CDA-MET	1	1.6	0.04–8.8	
	RMP-TET	1	1.6	0.04–8.8	
Three drugs	AMP-CDA-MET	4	6.5	1.8–15.9	Yes
	AMP-CDA-RMP	1	1.6	0.04–8.8	Yes
	AMP-MET-OXA	2	3.3	0.4–11.3	
	AMP-RMP-TET	1	1.6	0.04–8.8	Yes
	AMP-RMP-SXT	1	1.6	0.04–8.8	Yes
	AMP-TET-TGC	1	1.6	0.04–8.8	Yes
	AMP-STP-SXT	1	1.6	0.04–8.8	Yes
Four drugs	AMP-CDA-MET-OXA	1	1.6	0.04–8.8	Yes
	AMP-MET-OXA-RMP	1	1.6	0.04–8.8	Yes
	AMP-MET-OXA-STP	1	1.6	0.04–8.8	Yes
	AMP-MET-RMP-SXT	1	1.6	0.04–8.8	Yes
Five drugs	AMP-CDA-MET-OXA-TGC	1	1.6	0.04–8.8	Yes
	AMP-CDA-MET-RMP-STP	1	1.6	0.04–8.8	Yes
	AMP-MET-OXA-RMP-STP	1	1.6	0.04–8.8	Yes
Six drugs	AMP-CDA-MET-OXA-STP-TET	1	1.6	0.04–8.8	Yes
Seven drugs	AMP-CDA-GEN-MET-OXA-STP-TET	1	1.6	0.04–8.8	Yes
	AMP-CDA-MET-OXA-RMP-STP-TET	1	1.6	0.04–8.8	Yes

AMP, ampicillin; CDA, clindamycin; GEN, gentamicin; MET, methicillin; OXA, oxacillin; RMP, rifampicin; STP, streptomycin; SXT, sulfamethoxazole-trimethoprim; TET, tetracycline; TGC, Tigecycline.

### 3.5. Inter-species interaction in different locations

We observed direct and indirect interactions between macaques and people, cattle, goats, and dogs (Table 6). The most frequently observed inter-species interactions were between people and macaques (Table 6). We observed the highest number of human-macaque contacts in Gendaria, Dhaka, with 40 observations of direct contact and 204 observations of indirect contact. In contrast, Monohardi and Narshingdi had the lowest number of observed direct (12) and indirect (76) contact occurrences. No direct contact was observed between macaques, goats, and cattle in Gendaria and Rothkhola in Dhaka. No direct or indirect contact was observed between macaques and any domestic animals in Rajendropur, Gazipur (Table 6).

**Table 6:** Direct and indirect interactions between macaques and other species (8 h).

**Locations**	**Direct contact**				**Indirect contact**			
	**Human-Macaque**	**Goat-Macaque**	**Cattle-Macaque**	**Dog-Macaque**	**Human-Macaque**	**Goat-Macaque**	**Cattle-Macaque**	**Dog-Macaque**
Gendaria	40 (24.7%)	0	0	7 (10%)	204 (22.1%)	0	0	27 (15%)
Rothkhola	21 (13%)	0	0	3 (4.3%)	157 (17%)	9 (5%)	0	14 (7.8%)
Dhamrai	27 (16.7%)	9 (16.3%)	3 (13%)	22 (31.4%)	109 (11.8%)	35 (19.7%)	13 (11.7%)	43 (23.9%)
Bormi	18 (11.1%)	14 (25.5%)	6 (26%)	22 (31.4%)	108 (11.7%)	50 (28.1%)	38 (34.2%)	50 (27.8%)
Rajendropur	18 (11.1%)	0	0	0	104 (11.3%)	0	0	0
Charmuguria	26 (16%)	26 (47.2%)	10 (43.7%)	10 (14.3%)	164 (17.8%)	50 (28.1%)	46 (41.5%)	34 (18.9%)
Monohardi	12 (7.4%)	6 (10.9%)	4 (17.3%)	6 (8.6%)	76 (8.3%)	34 (19.1%)	14 (12.6%)	12 (6.6%)
**Total**	162	55	23	70	922	178	111	180

The contact rate between macaques and people was high in urban habitats for both direct contacts per hour (3) and indirect contacts per hour (22) (Table 7).

**Table 7:** Contact rate of inter-species interaction.

**Habitat**	**Inter-species interaction**	**Direct contacts /hour**	**Indirect contacts /hour**
Urban	Human-Macaque	3	22
	Goat-Macaque	0	1
	Cattle-Macaque	0	0
	Dog-Macaque	1	3
Peri-urban	Human-Macaque	3	13
	Goat-Macaque	1	4
	Cattle-Macaque	1	2
	Dog-Macaque	2	4
Rural	Human-Macaque	1	15
	Goat-Macaque	4	5
	Cattle-Macaque	1	4
	Dog-Macaque	1	3

## 4. Discussion

We found evidence of antimicrobial-resistant bacteria in free-ranging macaque populations. This suggests that macaques are infected through contact with infected people or animals or environmental contamination. We observed direct and indirect contact between macaques and people and domestic animals at all sites where samples were collected, providing a potential transmission pathway for antimicrobial resistant bacteria, especially considering that free-ranging macaques in Bangladesh are not treated with antimicrobials.

We found that the prevalence of *Salmonella* spp. was low (5%). Several other studies have found similarly low rates of salmonella infection in other species of non-human primates, including a prevalence of 3% in *Salmonella* spp. in captive primate populations at the national center for primate biology (48). A longitudinal study in Thailand also found a similar prevalence of (7%) *Salmonella* serotypes in captive wildlife, including non-human primates (49). Another study from Bwindi and Mgahinga in Uganda reported that 13% of free-range mountain gorillas (*Gorilla gorilla beringei*) had *Salmonella* spp. (27).

We found that the macaques we sampled had *Salmonella* spp. most frequently resistant to tetracycline (89%). This is in line with a previous study where 95% of *Salmonella* sp. collected samples from south America, Africa, and Southeast Asia to the National Center for Primate Biology were resistant to tetracycline (48). Other studies found lower levels of tetracycline resistance in *Salmonella* spp. isolated from captive wildlife (29%) in Trinidad, which contains mammals (21%), birds (25%), and reptiles (40%) (49) and non-human primates (38.3%) in the USA (50). It may be that the prevalence of tetracycline resistant *Salmonella* spp. is associated with the usage of tetracycline to treat livestock. In Bangladesh, tetracycline is commonly used to treat livestock and does not require a prescription. Salmonella resistance to azithromycin was the second highest as it was detected in 83% of *Salmonella* spp. isolates in the present study. A previous study in Bangladesh found similarly high (95%) rates of azithromycin resistance *Salmonella* spp. in human patients (51). The authors proposed that the high rate of azithromycin resistance in *Salmonella* spp. may be due to the frequent misuse of this antibiotic to treat people for enteric fever and the common cold and possible transmission to macaques *via* environmental contamination through excrement (52). In addition, 50% of *Salmonella* spp. samples were also found to be resistant to sulfamethoxazole-trimethoprim. This result differs slightly with a recent study conducted in Bangladesh, in which 68 and 58% of *Salmonella enterica Serovar Typhi* samples were resistant to sulfamethoxazole and trimethoprim, respectively (53). In our investigation, the prevalence of nalidixic acid-resistant *Salmonella* was 44%, which is lower than the prevalence reported in several previous studies of human patients (73–93%) (53–55). Few other AMR studies have been conducted in other wild species (wild birds, squirrels, deer) in Bangladesh and found higher antimicrobial resistance in *Salmonella, E. coli*, and *Staphylococcus* (14, 56, 57).

We also found that *Salmonella* spp. isolates were susceptible to ciprofloxacin, gentamicin, and imipenem. These findings were similar to those reported by previous studies in which *Salmonella* spp. isolated from human samples in Montenegro, Bangladesh, and India were sensitive to ciprofloxacin (99.5%) (58), gentamicin (100%) (59), imipenem (100%) (60), respectively.

We found the *Staphylococcus* spp. prevalence was 16%, which was much lower than a previous study (61%) on non-human primates in Africa (24). In our study, the prevalence of resistant *Staphylococcus* spp. was higher (15%) than that of *Salmonella* spp. However, it was much lower than what was detected in *Macaca mulatta* in Spain (80%) (61), the Netherlands (39%) (25), and Wisconsin, USA (23.6%) (26). We found that *Staphylococcus* spp. in rhesus macaques was highly resistant (93%) to ampicillin, which contrasts a study that found that *S. aureus* isolates from African non-human primates was 2.9% resistant to penicillin (24). The increased interaction between synanthropic rhesus macaques and humans in South Asia is likely contributing to the higher ampicillin resistance and transfer of resistance genes in South Asia compared to African regions (62). Taylor and Grady reported 75% resistance to methicillin/oxacillin in *Staphylococcus* spp. *in vitro* (30).

*Staphylococcus* spp. were highly sensitive to chloramphenicol, gentamycin, linezolid, oxacillin, tigecycline, and trimethoprim-sulfamethoxazole in our study. This sensitivity is similar to that found in human clinical specimens in the USA, where more than 90% of multidrug-resistant *Staphylococcus* spp. were sensitive to trimethoprim-sulfamethoxazole, linezolid, and vancomycin (63). The higher sensitivity of *Staphylococcus* spp. to these antimicrobials may be due to infection of macaque-specific *Staphylococcus aureus* (25). Another reason may be infrequent exposure of humans and animals to chloramphenicol, linezolid, Oxacillin, etc. (64).

Rhesus macaques from the Dhaka District had a higher odd (OR = 11.1) of harboring resistant *Staphylococcus* spp. We suspect this may be associated with the high human population density levels in Dhaka City and improper waste disposal management (65). Moreover, household drinking water supply contains diversified microorganisms that easily increase the chance of getting resistant organisms (17). A similar reason may be corresponded with the higher odds of resistance to *Salmonella* spp. in the urban areas compared to the rural areas. Macaques from the peri-urban areas also had a high odd (OR = 5.6) of having resistant *Staphylococcus* spp. Peri-urban regions have a decentralized waste management system (66) and transmission of pathogenic bacteria into the river through sewage systems (67, 68). As in the peri-urban area, rural areas were more likely to experience Staphylococcus resistance, possibly due to the transmission of pathogenic bacteria into rural *via* the river; additionally, waste disposal is poor in the rural area.

The age-dependent differences in AMR can also be useful for monitoring the trend of antibiotic-resistant strains spreading at high-risk interfaces in Bangladesh. In reality, the behavior of individuals of the same species can vary depending on their age and gender. It is believed that young are less exposed to antibiotics than adults throughout their lifetimes, and younger animals may therefore have a lower prevalence of AMR isolates than older animals. However, our results revealed higher odds of Staphylococcus resistance in the sub-adult compared to the adult. On the other hand, no significant differences in relation to animal age for Salmonella resistance. Similar to this study, the insignificant difference in MDR has been reported in wild micromammals from Northern, Italy (69). The age-dependent AMR phenomena in cattle and pigs are detected regardless of geographic region and production methods. However, the nature of the AMR phenomenon is currently unexplained (70).

We discovered that macaques and people had more direct and indirect contact per hour in urban and peri-urban areas. These are most likely significant risk factors for transmitting resistant organisms/genes from humans to macaques and vice versa. Microorganism transmission from humans to non-human primates has been observed for approximately three decades (71) and happening frequently e.g., SARS-CoV-2 infection in a gorilla at a zoo in California (72). A recent qualitative study suggests that interviewees from some of these regions in Bangladesh believe that recent human encroachment into the natural habitat of macaques may have increased the amount of contact between people and macaques (73). Hasan et al. reported that the distribution of macaques is high in densely populated urban areas, such as old Dhaka (23). Macaques can also be found in temples and shrines where people frequently leave food (34). As a result, the rhesus macaque may acquire resistant microorganisms from humans. As expected, the contact rate between livestock and macaques was higher in rural areas, given the abundance of livestock in these areas. According to one study, many disease-causing bacteria in livestock in Bangladesh were resistant to available antibiotics, most likely due to improper antimicrobial use (3). Contact between livestock and macaque can potentially transmit resistant organisms and/or genes. Further, macaques frequently take waste from dustbins, snatch household food and clothes, and damage crops (74). Our study detected that interaction between livestock, dogs, and people with macaques frequently occurs during the conflict, crop raiding, and when people provide food (75). Stray dogs eat waste from dustbins and the roadside and are infected with microorganisms resistant to the majority of commonly used antibiotics (76).

The exceedingly high rates of AMR that we found in *Salmonella* spp. (100%) and *Staphylococcus* spp. (95%) suggest that macaque exposure to antimicrobial resistant bacteria at all the sampling sites was likely very high. Antibiotics are commonly used in treating humans, livestock, and poultry in Bangladesh; therefore, antibiotic residues and resistant organisms could have been passed through environmental sources and/or interspecies interaction with wild species. Rhesus macaque and other wildlife are rarely treated with antibiotics, but present study showed a relatively high frequency of antimicrobial resistance in *Salmonella* and *Staphylococcus* in the rhesus macaque. So, Nationwide programs are important to control the irrational use of antibiotics and improve the waste management and sewage system and reduce human-wildlife and livestock-wildlife interaction to decrease antimicrobial resistance among humans and animals in the future.

## 5. Conclusions

AMR is a global concern and public health threat, and the fact that it is spilling over into wildlife indicates potential widespread environmental contamination. The study identified the prevalence of 5% *Salmonella* spp. and 16% *Staphylococcus* spp. in rhesus macaque, nearly all of which were resistant (100 and 95%, respectively) in different locations in Bangladesh. The isolated organisms showed various levels of resistance to different antimicrobials. Multidrug resistance patterns in *Salmonella* spp. and *Staphylococcus* spp. were identified, including cultures resistant to up to seven antimicrobials. The high level of resistance could be attributed to interactions and transmission of resistant microorganisms between macaques, humans, and livestock. Human-macaque interactions were more common in urban habitats, while livestock-macaque interactions were more common in peri-urban and rural habitats. Even though rhesus macaques are not directly treated with antimicrobials, this study found an alarming level of resistant organisms in macaques. These findings should be viewed as an indicator of significant environmental contamination and an urgent call for stricter controls of the use of antimicrobials.

## Data availability statement

The original contributions presented in the study are included in the article/supplementary material, further inquiries can be directed to the corresponding author.

## Ethics statement

The animal study was reviewed and approved by Animal Experimentation Ethics Committee (AEEC) [permit number: CVASU/Dir (R&E) AEEC/2015/751], Chattogram Veterinary and Animal Sciences University, Chattogram, Bangladesh.

## Author contributions

MRa, AI, MRo, JE, and MH: conceptualization. MRa, AI, and MU: methodology. MRa and AI: software, formal analysis, and writing—original draft preparation. AI, SI, MRa, EH, MS, MRo, JE, and MH: validation. AI and MH: investigation. MRa, AI, and MH: resources. MRa, SI, and MU: data curation. AI, SI, MRa, EH, MRo, MS, and MH: writing—review and editing. MU and MS: laboratory support. AI, MF, and MH: supervision and project administration. JE and AI: funding acquisition. All authors have read and agreed to the published version of the manuscript.
